# Supplementary material for: Methaemoglobin as a surrogate marker of primaquine antihypnozoite activity in Plasmodium vivax malaria: A systematic review and individual patient data meta-analysis
Source: PLoS Med. 2024 Sep 27;21(9):e1004411. doi: 10.1371/journal.pmed.1004411 (PMC11469483; doi:10.1371/journal.pmed.1004411)
Supplement: S1 Text — Systematic search terms for the databases. Signalling questions for risk of bias assessment using the QUIPS tool adapted to the current analysis. Table A. PRISMA-IPD checklist. Table B. Risk of bias assessment. Table C. Studies included in analysis. Table D. Studies that were eligible for analysis but not included in the pooled data. Table E. Comparison of characteristics of patients (as originally reported) who received primaquine between included and eligible but not included studies. Table F. Regression table output for the main Cox proportional hazards model. Fig A. Study sites that contributed to the pooled data in this individual patient data meta-analysis. Fig B. Distribution of weight-adjusted primaquine daily dose by primaquine regimen. Fig C. Dynamics of primaquine-induced increases in blood methaemoglobin over time. Fig D. Day 7 methaemoglobin concentrations by primaquine regimen and dose group. Fig E. Inverse J-shaped association between patient age and day 7 methaemoglobin by recurrence status, after controlling for daily mg/kg primaquine dose. Fig F. Distribution of day 7 methaemoglobin among the patients on (A) the original scale and (B) the logarithmic scale. Fig G. Mixed-effects estimates for the intercept and slope (mean difference). Fig H. Mixed-effects estimates for the slope (hazard ratio). Fig I. Example of sample size calculations for future studies. Fig J. Comparisons of estimates from the current analysis with those previous studies. (DOCX) [file pmed.1004411.s001.docx]

# Supporting Information

[**Systematic search terms for the databases 2**](#_Toc174983235)

[**Signalling questions for risk of bias assessment using the QUIPS tool adapted to the current analysis 8**](#_Toc174983236)

[**Table A. PRISMA-IPD checklist 3**](#_Toc174984278)

[**Table B. Risk of bias assessment 9**](#_Toc174984279)

[**Table C. Studies included in analysis 11**](#_Toc174984280)

[**Table D. Studies that were eligible for analysis but not included in the pooled data 13**](#_Toc174984281)

[**Table E. Comparison of characteristics of patients (as originally reported) who received primaquine between included and eligible but not included studies 14**](#_Toc174984282)

[**Table F. Regression table output for the main Cox proportional hazards model 17**](#_Toc174984283)

[**Figure A. Study sites that contributed to the pooled data in this individual patient data meta-analysis 10**](#_Toc174984285)

[**Figure B. Distribution of weight-adjusted primaquine daily dose by primaquine regimen 12**](#_Toc174984286)

[**Figure C. Dynamics of primaquine-induced increases in blood methaemoglobin over time 15**](#_Toc174984287)

[**Figure D. Day 7 methaemoglobin concentrations by primaquine regimen and dose group 15**](#_Toc174984288)

[**Figure E. Inverse J-shaped association between patient age and day 7 methaemoglobin by recurrence status, after controlling for daily mg/kg primaquine dose 16**](#_Toc174984289)

[**Figure F. Distribution of day 7 methaemoglobin among the patients on (A) the original scale and (B) the logarithmic scale 16**](#_Toc174984290)

[**Figure G. Mixed-effects estimates for the intercept and slope (mean difference) 17**](#_Toc174984291)

[**Figure H. Mixed-effects estimates for the slope (hazard ratio) 18**](#_Toc174984292)

[**Figure I. Example of sample size calculations for future studies 19**](#_Toc174984293)

[**Figure J. Comparisons of estimates from the current analysis with those previous studies 19**](#_Toc174984294)

**1Systematic search terms for the databases**

Vivax AND (artefenomel OR arterolane OR amodiaquine OR atovaquone OR artemisinin OR arteether OR artesunate OR artemether OR artemotil OR azithromycin OR artekin OR chloroquine OR chlorproguanil OR cycloguanil OR clindamycin OR coartem OR dapsone OR dihydroartemisinin OR duo-cotecxin OR doxycycline OR halofantrine OR lumefantrine OR lariam OR malarone OR mefloquine OR naphthoquine OR naphthoquinone OR piperaquine OR primaquine OR proguanil OR pyrimethamine OR pyronaridine OR proguanil OR quinidine OR quinine OR riamet OR sulphadoxine OR tetracycline OR tafenoquine)

**Table A. PRISMA-IPD checklist**

| **PRISMA-IPD**  **Section/topic** | **Item No** | **Checklist item** | **Reported on page** |
| --- | --- | --- | --- |
| **Title** | | | |
| Title | 1 | Identify the report as a systematic review and meta-analysis of individual participant data. | Title |
| **Abstract** | | | |
| Structured summary | 2 | Provide a structured summary including as applicable: | Abstract |
|  |  | **Background**: state research question and main objectives, with information on participants, interventions, comparators and outcomes. |  |
|  |  | **Methods**: report eligibility criteria; data sources including dates of last bibliographic search or elicitation, noting that IPD were sought; methods of assessing risk of bias. |  |
|  |  | **Results**: provide number and type of studies and participants identified and number (%) obtained; summary effect estimates for main outcomes (benefits and harms) with confidence intervals and measures of statistical heterogeneity. Describe the direction and size of summary effects in terms meaningful to those who would put findings into practice. |  |
|  |  | **Discussion:** state main strengths and limitations of the evidence, general interpretation of the results and any important implications. |  |
|  |  | **Other:** report primary funding source, registration number and registry name for the systematic review and IPD meta-analysis. |  |
| **Introduction** | | | |
| Rationale | 3 | Describe the rationale for the review in the context of what is already known. | Introduction, paragraphs 1–4 |
| Objectives | 4 | Provide an explicit statement of the questions being addressed with reference, as applicable, to participants, interventions, comparisons, outcomes and study design (PICOS). Include any hypotheses that relate to particular types of participant-level subgroups. | Introduction, paragraph 4 |
| **Methods** | | | |
| Protocol and registration | 5 | Indicate if a protocol exists and where it can be accessed. If available, provide registration information including registration number and registry name. Provide publication details, if applicable. | Methods, paragraph 2 |
| Eligibility criteria | 6 | Specify inclusion and exclusion criteria including those relating to participants, interventions, comparisons, outcomes, study design and characteristics (e.g. years when conducted, required minimum follow-up). Note whether these were applied at the study or individual level i.e. whether eligible participants were included (and ineligible participants excluded) from a study that included a wider population than specified by the review inclusion criteria. The rationale for criteria should be stated. | Methods, paragraphs 1–3 |
| Identifying studies - information sources | 7 | Describe all methods of identifying published and unpublished studies including, as applicable: which bibliographic databases were searched with dates of coverage; details of any hand searching including of conference proceedings; use of study registers and agency or company databases; contact with the original research team and experts in the field; open adverts and surveys. Give the date of last search or elicitation. | Methods, paragraph 1 |
| Identifying studies - search | 8 | Present the full electronic search strategy for at least one database, including any limits used, such that it could be repeated. | Methods, paragraph 1; Supporting Information |
| Study selection processes | 9 | State the process for determining which studies were eligible for inclusion. | Methods, paragraphs 1–3 |
| Data collection processes | 10 | Describe how IPD were requested, collected and managed, including any processes for querying and confirming data with investigators. If IPD were not sought from any eligible study, the reason for this should be stated (for each such study). | Methods, paragraph 1–3 |
|  |  | If applicable, describe how any studies for which IPD were not available were dealt with. This should include whether, how and what aggregate data were sought or extracted from study reports and publications (such as extracting data independently in duplicate) and any processes for obtaining and confirming these data with investigators. |  |
| Data items | 11 | Describe how the information and variables to be collected were chosen. List and define all study level and participant level data that were sought, including baseline and follow-up information. If applicable, describe methods of standardising or translating variables within the IPD datasets to ensure common scales or measurements across studies. | Methods, paragraphs 3–10 |
| IPD integrity | A1 | Describe what aspects of IPD were subject to data checking (such as sequence generation, data consistency and completeness, baseline imbalance) and how this was done. | Methods, paragraphs 1–14 |
| Risk of bias assessment in individual studies. | 12 | Describe methods used to assess risk of bias in the individual studies and whether this was applied separately for each outcome. If applicable, describe how findings of IPD checking were used to inform the assessment. Report if and how risk of bias assessment was used in any data synthesis. | Methods, paragraph 14 |
| Specification of outcomes and effect measures | 13 | State all treatment comparisons of interests. State all outcomes addressed and define them in detail. State whether they were pre-specified for the review and, if applicable, whether they were primary/main or secondary/additional outcomes. Give the principal measures of effect (such as risk ratio, hazard ratio, difference in means) used for each outcome. | Methods, paragraphs 1–14 |
| Synthesis methods | 14 | Describe the meta-analysis methods used to synthesise IPD. Specify any statistical methods and models used. Issues should include (but are not restricted to):   - Use of a one-stage or two-stage approach. - How effect estimates were generated separately within each study and combined across studies (where applicable). - Specification of one-stage models (where applicable) including how clustering of patients within studies was accounted for. - Use of fixed or random effects models and any other model assumptions, such as proportional hazards. - How (summary) survival curves were generated (where applicable). - Methods for quantifying statistical heterogeneity (such as I^2^ and t^2^). - How studies providing IPD and not providing IPD were analysed together (where applicable). - How missing data within the IPD were dealt with (where applicable). | Methods, paragraphs 1–14 |
| Exploration of variation in effects | A2 | If applicable, describe any methods used to explore variation in effects by study or participant level characteristics (such as estimation of interactions between effect and covariates). State all participant-level characteristics that were analysed as potential effect modifiers, and whether these were pre-specified. | Methods, paragraphs 10–14 |
| Risk of bias across studies | 15 | Specify any assessment of risk of bias relating to the accumulated body of evidence, including any pertaining to not obtaining IPD for particular studies, outcomes or other variables. | Methods, paragraph 1; Supporting Information |
| Additional analyses | 16 | Describe methods of any additional analyses, including sensitivity analyses. State which of these were pre-specified. | Methods, paragraphs 11–12 |
| **Results** | | | |
| Study selection and IPD obtained | 17 | Give numbers of studies screened, assessed for eligibility, and included in the systematic review with reasons for exclusions at each stage. Indicate the number of studies and participants for which IPD were sought and for which IPD were obtained. For those studies where IPD were not available, give the numbers of studies and participants for which aggregate data were available. Report reasons for non-availability of IPD. Include a flow diagram. | Results, paragraph 1 |
| Study characteristics | 18 | For each study, present information on key study and participant characteristics (such as description of interventions, numbers of participants, demographic data, unavailability of outcomes, funding source, and if applicable duration of follow-up). Provide (main) citations for each study. Where applicable, also report similar study characteristics for any studies not providing IPD. | Results, paragraph 2; Supporting Information |
| IPD integrity | A3 | Report any important issues identified in checking IPD or state that there were none. | Results, paragraph 1 |
| Risk of bias within studies | 19 | Present data on risk of bias assessments. If applicable, describe whether data checking led to the up-weighting or down-weighting of these assessments. Consider how any potential bias impacts on the robustness of meta-analysis conclusions. | Results, paragraph 1; Supporting Information |
| Results of individual studies | 20 | For each comparison and for each main outcome (benefit or harm), for each individual study report the number of eligible participants for which data were obtained and show simple summary data for each intervention group (including, where applicable, the number of events), effect estimates and confidence intervals. These may be tabulated or included on a forest plot. | Results, paragraph 7 |
| Results of syntheses | 21 | Present summary effects for each meta-analysis undertaken, including confidence intervals and measures of statistical heterogeneity. State whether the analysis was pre-specified, and report the numbers of studies and participants and, where applicable, the number of events on which it is based. | Results, paragraphs 6–8 |
|  |  | When exploring variation in effects due to patient or study characteristics, present summary interaction estimates for each characteristic examined, including confidence intervals and measures of statistical heterogeneity. State whether the analysis was pre-specified. State whether any interaction is consistent across trials. |  |
|  |  | Provide a description of the direction and size of effect in terms meaningful to those who would put findings into practice. |  |
| Risk of bias across studies | 22 | Present results of any assessment of risk of bias relating to the accumulated body of evidence, including any pertaining to the availability and representativeness of available studies, outcomes or other variables. | Results, paragraph 1; Supporting Information |
| Additional analyses | 23 | Give results of any additional analyses (e.g. sensitivity analyses). If applicable, this should also include any analyses that incorporate aggregate data for studies that do not have IPD. If applicable, summarise the main meta-analysis results following the inclusion or exclusion of studies for which IPD were not available. | Results, paragraphs 6­–8; Supporting Information |
| **Discussion** | | | |
| Summary of evidence | 24 | Summarise the main findings, including the strength of evidence for each main outcome. | Discussion, paragraphs 1–3 |
| Strengths and limitations | 25 | Discuss any important strengths and limitations of the evidence including the benefits of access to IPD and any limitations arising from IPD that were not available. | Discussion, paragraphs 2–8 |
| Conclusions | 26 | Provide a general interpretation of the findings in the context of other evidence. | Discussion, paragraph 9 |
| Implications | A4 | Consider relevance to key groups (such as policy makers, service providers and service users). Consider implications for future research. | Discussion, paragraph 9 |
| **Funding** | | | |
| Funding | 27 | Describe sources of funding and other support (such as supply of IPD), and the role in the systematic review of those providing such support. | Discussion, paragraph 11 |

**2****Signalling questions for risk of bias assessment using the QUIPS tool adapted to the current analysis**

Domain 1: The study sample represents the population of interest on key characteristics, sufficient to limit potential bias of the observed relationship between the predictive factor and outcome.

- The source population or population of interest is adequately described.
- The baseline study sample (i.e., individuals entering the study) is adequately described.
- The sampling frame and recruitment are adequately described, including methods to identify the sample sufficient to limit potential bias.
- Period of recruitment is adequately described.
- Place of recruitment (setting, level of endemicity, geographic location) are adequately described.
- Inclusion and exclusion criteria are adequately described).

Domain 2: Loss to follow up (from baseline sample to study population analysed) is not associated with certain characteristics (i.e., the study data adequately represent the sample) sufficient to limit potential bias to the observed relationship between predictive factor and outcome.

- Response rate (i.e., proportion of study sample completing the study and providing outcome data) is adequate.
- Attempts to collect information on participants who dropped out of the study are described.
- Reasons for loss to follow up are provided.
- Participants lost to follow up are adequately described.
- There are no important differences between participants who completed the study and those who did not.

Domain 3: Predictive factor and drug intervention are adequately measured in study participants to sufficiently limit potential bias.

- A clear definition or description of the primaquine regimen and measured methaemoglobin is provided (e.g., including dose, level, duration of exposure, and clear specification of the method of measurement).
- Adequately accurate and reliable measurement of primaquine doses and methaemoglobin concentrations to limit misclassification bias.
- Continuous variables are reported, or clinically relevant cut points (i.e., not data-dependent) are used.
- Method and setting of methaemoglobin measurement are the same for all study participants.
- Adequate proportion of the study sample has complete methaemoglobin data.
- Adequate adherence or supervision of primaquine administration.

Domain 4: Outcome of interest is adequately measured in study participants to sufficiently limit potential bias.

- A clear definition of the outcome is provided, including duration of follow up.
- Method of outcome measurement used is adequately accurate and reliable to limit misclassification bias.
- Method and setting of outcome measurement are the same for all study participants.

**Table B. Risk of bias assessment**

| **Study** | **QUIPS domain** | | | |
| --- | --- | --- | --- | --- |
|  | **Study participant** | **Study attrition** | **Predictive factor measurement** | **Outcome measurement** |
| Pasaribu 2013 [1] | Low | Low | Low | Low |
| Sutanto 2013 [2] | Low | Low | Low | Low |
| Llanos-Cuentas 2014 [3] | Low | Low | Moderate | Low |
| Nelwan 2015 [4] | Low | Low | Low | Low |
| Chu 2019 [5] | Low | Low | Low | Low |
| Lacerda 2019 [6] | Low | Moderate | Moderate | Low |
| Llanos-Cuentas 2019 [7] | Low | Low | Low | Low |
| Taylor 2019 [8] | Low | Moderate | Moderate | Low |

**
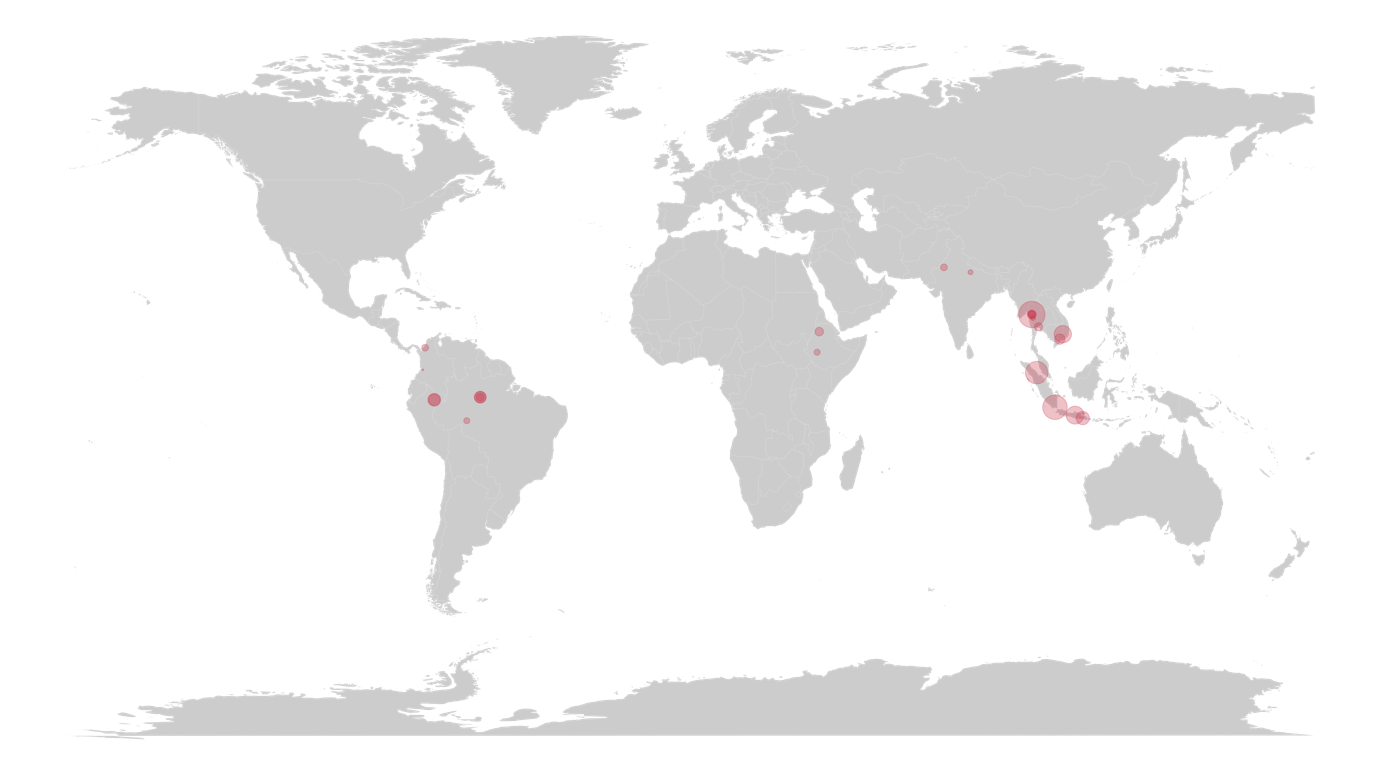
**

**Figure A. Study sites that contributed to the pooled data in this individual patient data meta-analysis**

Red bubble represents a study site with size proportional to the square root of the number of patients. Basemap shapefile data obtained from the public domain Natural Earth project (https://www.naturalearthdata.com), accessed via the open-source R maps [9] and ggplot2 [10] packages.

**Table C. Studies included in analysis**

| **Paper** | **Study site** | **Country** | **Region** | **Latitude** | **Longitude** | **Year start** | **Year end** | **MAP incidence rate (per 1000 persons)** | **Transmission intensity#** | **Relapse periodicity§** |
| --- | --- | --- | --- | --- | --- | --- | --- | --- | --- | --- |
| **Pasaribu 2013 [1]** | Tanjung Leidong | Indonesia | Asia-Pacific | 2.77 | 99.98 | 2011 | 2011 | 2.75 | Moderate | High |
| **Sutanto 2013 [2]** | Lumajang | Indonesia | Asia-Pacific | -8.13 | 113.22 | 2010 | 2011 | 36.88* | High | High |
| **Llanos-Cuentas 2014 [3]** | Bangkok | Thailand | Asia-Pacific | 13.76 | 100.50 | 2011 | 2013 | 0.16 | Low | High |
| **Llanos-Cuentas 2014 [3]** | Mae Sot | Thailand | Asia-Pacific | 16.72 | 98.58 | 2011 | 2013 | 3.07 | Moderate | High |
| **Llanos-Cuentas 2014 [3]** | Lucknow | India | Asia-Pacific | 26.85 | 80.95 | 2011 | 2013 | 2.84 | Moderate | Low |
| **Llanos-Cuentas 2014 [3]** | Bikaner | India | Asia-Pacific | 28.02 | 73.31 | 2011 | 2013 | 2.85 | Moderate | Low |
| **Llanos-Cuentas 2014 [3]** | Iquitos | Peru | Americas | -3.74 | -73.25 | 2011 | 2013 | 40.49 | High | Low |
| **Llanos-Cuentas 2014 [3]** | Manaus | Brazil | Americas | -3.12 | -60.02 | 2011 | 2013 | 42.81 | High | Low |
| **Nelwan 2015 [4]** | Sragen | Indonesia | Asia-Pacific | -7.42 | 111.02 | 2013 | 2013 | 42.44* | High | High |
| **Chu 2019 [5]** | Mae Sot | Thailand | Asia-Pacific | 16.72 | 98.58 | 2012 | 2014 | 3.09 | Moderate | High |
| **Lacerda 2019 [6]** | Manaus | Brazil | Americas | -3.12 | -60.02 | 2013 | 2016 | 41.14 | High | Low |
| **Lacerda 2019 [6]** | Porto Velho | Brazil | Americas | -8.76 | -63.90 | 2013 | 2016 | 9.23 | Moderate | Low |
| **Lacerda 2019 [6]** | Jimma | Ethiopia | Africa | 7.67 | 36.84 | 2013 | 2016 | 40.53 | High | Low |
| **Lacerda 2019 [6]** | Gondar | Ethiopia | Africa | 12.60 | 37.45 | 2013 | 2016 | 6.72 | Moderate | Low |
| **Lacerda 2019 [6]** | Mae Sot | Thailand | Asia-Pacific | 16.72 | 98.58 | 2013 | 2016 | 0.64 | Low | High |
| **Llanos-Cuentas 2019 [7]** | Manaus | Brazil | Americas | -3.12 | -60.02 | 2015 | 2016 | 18.55 | High | Low |
| **Llanos-Cuentas 2019 [7]** | Monteira | Colombia | Americas | 8.75 | -75.88 | 2015 | 2016 | 5.36 | Moderate | Low |
| **Llanos-Cuentas 2019 [7]** | Cali | Colombia | Americas | 3.45 | -76.53 | 2015 | 2016 | 1.93 | Moderate | Low |
| **Llanos-Cuentas 2019 [7]** | Iquitos | Peru | Americas | -3.74 | -73.25 | 2015 | 2016 | 58.05 | High | Low |
| **Llanos-Cuentas 2019 [7]** | Umphang | Thailand | Asia-Pacific | 15.88 | 98.92 | 2015 | 2016 | 1.08 | Moderate | High |
| **Llanos-Cuentas 2019 [7]** | Mae Sot | Thailand | Asia-Pacific | 16.72 | 98.58 | 2015 | 2016 | 1.08 | Moderate | High |
| **Llanos-Cuentas 2019 [7]** | Ho Chi Minh City | Vietnam | Asia-Pacific | 10.82 | 106.63 | 2015 | 2016 | 0.01 | Low | High |
| **Taylor 2019 [8]** | Dak O | Vietnam | Asia-Pacific | 12.00 | 107.50 | 2015 | 2017 | 0.24 | Low | High |
| **Taylor 2019 [8]** | Hanura | Indonesia | Asia-Pacific | -5.53 | 105.24 | 2015 | 2017 | 1.01 | Moderate | High |

MAP malaria atlas project. § Relapse periodicity was categorised as high (median relapse periodicity of 47 days or less) and low (median relapse periodicity of more than 47) [11]; # Transmission intensity was categorised as low (<1 case per 1000 person-years), moderate (1 case to <10 cases per 1000 person-years), and high (≥10 cases per 1000 person-years) according to subnational malaria incidence estimates for the median year of study enrolment [12]. *Based on the location where patients were infected by *P. vivax* in Indonesian Papua.

**
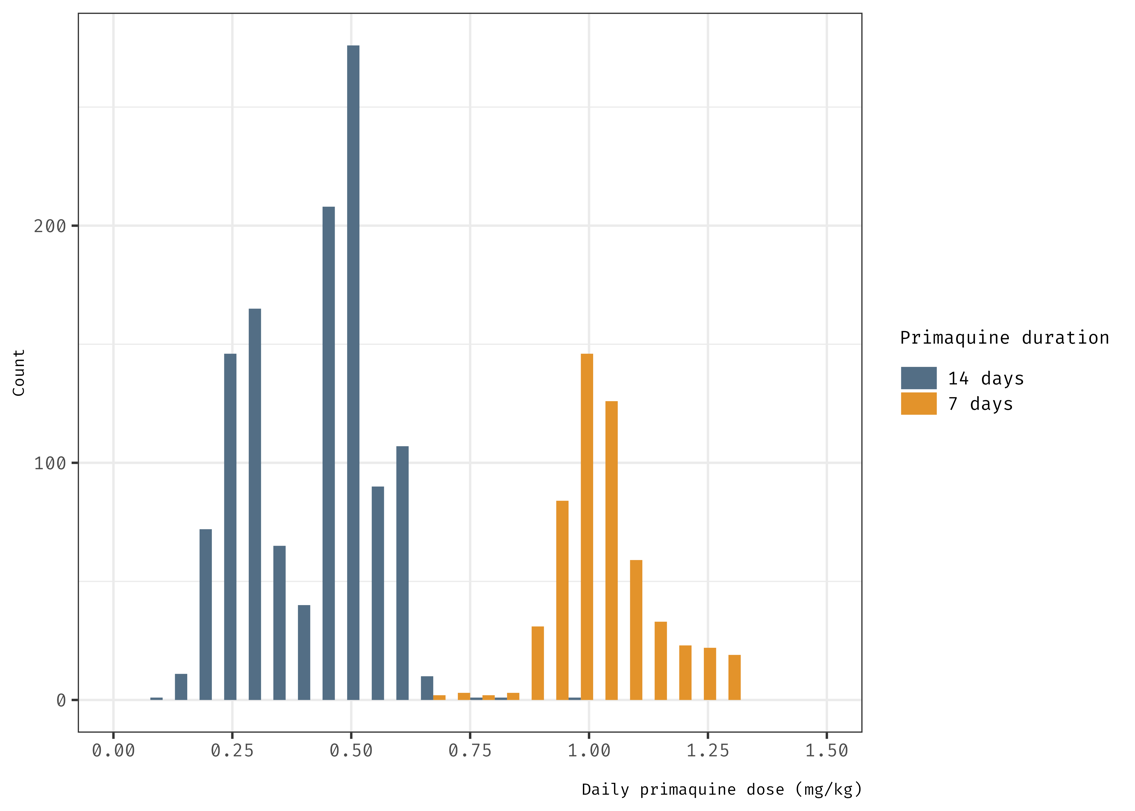
**

**Figure B. Distribution of weight-adjusted primaquine daily dose by primaquine regimen**

In the 14-day primaquine regimen, the observed two peaks reflect the targeted total primaquine dose of 3.5 and 7 mg (base) per kg body weight.

**Table D. Studies that were eligible for analysis but not included in the pooled data**

| **Characteristic** | **Study** | | | | | | |
| --- | --- | --- | --- | --- | --- | --- | --- |
|  | **Solari Soto [13]** | **Carmona-Fonseca [14]** | **Ley [15]** | **Fukuda [16]** | **Chu [17]** | **Moore [18]** | **Sutanto [19]** |
| **Year published** | 2002 | 2010 | 2016 | 2017 | 2018 | 2023 | 2023 |
| **Number of treatment arms** | 2 | 2 | 1 | 2 | 3 | 3 | 3 |
| **Number of sites** | 1 | 1 | 2 | 1 | 1 | 1 | 2 |
| **Region** | Americas | Americas | Asia-Pacific | Asia-Pacific | Asia-Pacific | Asia-Pacific | Asia-Pacific |
| **Country** | Peru | Colombia | Bangladesh | Thailand | Thailand | Papua New Guinea | Indonesia |
| **Follow-up (days)** | 60 | 120 | 28 | 120 | 365 | 63 | 180 |
| **Randomised** | Yes | Yes | No | Yes | Yes | No | Yes |
| **Recruitment period** | 1998­­–1999 | 2005­–2008 | 2014­–2015 | 2003–2005 | 2010­–2012 | 2013–2018 | 2018–2019 |
| **Treatment arms** | (1) Chloroquine + 14-day, low-dose primaquine,  (2) Chloroquine + 7-day, low-dose primaquine | (1) Chloroquine + 7-day, low-dose primaquine,  (2) Chloroquine + 3-day, low-dose primaquine | Chloroquine + 14-day, low-dose primaquine | (1) Chloroquine + 14-day, low-dose primaquine,  (2) Tafenoquine | (1) Artesunate,  (2) Chloroquine,  (3) Chloroquine + 14-day, high-dose primaquine | (1) Artemether-Lumefantrine + 14-day, high-dose primaquine  (2) Artemether-Lumefantrine + 7-day, high-dose primaquine  (3) Artemether-Lumefantrine + 3.5-day, high-dose primaquine | (1) Dihydroartemisinin-Piperaquine  (2) Dihydroartemisinin-Piperaquine + 14-day, low-dose primaquine  (3) Dihydroartemisinin-Piperaquine + tafenoquine |
| ***P. vivax* patients enrolled** | 60 | 79 | 66 | 70 | 644 | 71 | 150 |
| **Treated with primaquine** | 60 | 79 | 66 | 24 | 198 | 71 | 50 |
| **Supervision** | Yes | Yes | Yes | Yes | Yes | Yes | Yes |
| **Sex (primaquine receiving arm)** | 57% male | Mostly male | 62% male | 83% male | 64% male | 62% male | 100% male |
| **Age (in years, primaquine receiving arm)** | Average = 26.5 | Range = 10­–17 | Median = 18 (mono-infection), 14 (mixed) | Median = 30 | Median = 18 | Median = 6.8 | Mean = 28.8 |
| **Reason for exclusion** | No response from investigators | Data not available | Missing minimum data | Data not provided | Missing minimum data | Data not available by 22 September 2022 | Data not available by 22 September 2022 |

IPD individual patient data. Updated searches between 30 September 2022 and 26 July 2024 identified nine published studies of *P. vivax*.

**Table E. Comparison of characteristics of patients (as originally reported) who received primaquine between included and eligible but not included studies**

| **Characteristic** | **Included studies (n = 8)** | **Eligible but not included studies (n = 7)** |
| --- | --- | --- |
| **Region, studies (percentage)** | | |
| Asia-Pacific | 21 (58.3%)^€^ | 5 (71.4%) |
| Africa | 4 (11.1%)^€^ | 0 (0%) |
| The Americas | 11 (30.6%)^€^ | 2 (28.6%) |
| **Year of enrolment, studies (percentage)** | | |
| Pre-2015 | 6 (75%) | 5 (71.4%) |
| 2015-2019 | 2 (25%) | 2 (28.6%) |
| **Follow up duration in days, studies (percentage)** | | |
| 42 | 0 (0%) | 1 (14.3%) |
| >42 to <120 | 0 (0%) | 2 (28.6%) |
| 120 | 0 (0%) | 2 (28.6%) |
| >120 | 8 (100%) | 2 (28.6%) |
| **Age (weighted-average years)^#,$^** | 19.1 | 19.2^§^ |
| **Male (weighted percentage)^#^** | 65.8% | 67.3%^§^ |

€ Included multinational studies. Number and percentage are derived from the number of study sites, not studies, within each region. # Weights approximated by the numbers of vivax patients treated with primaquine. $ Study-specific average is the mean or median in vivax patients treated with primaquine. § Excluding one study for which the required summary statistics not available.

**
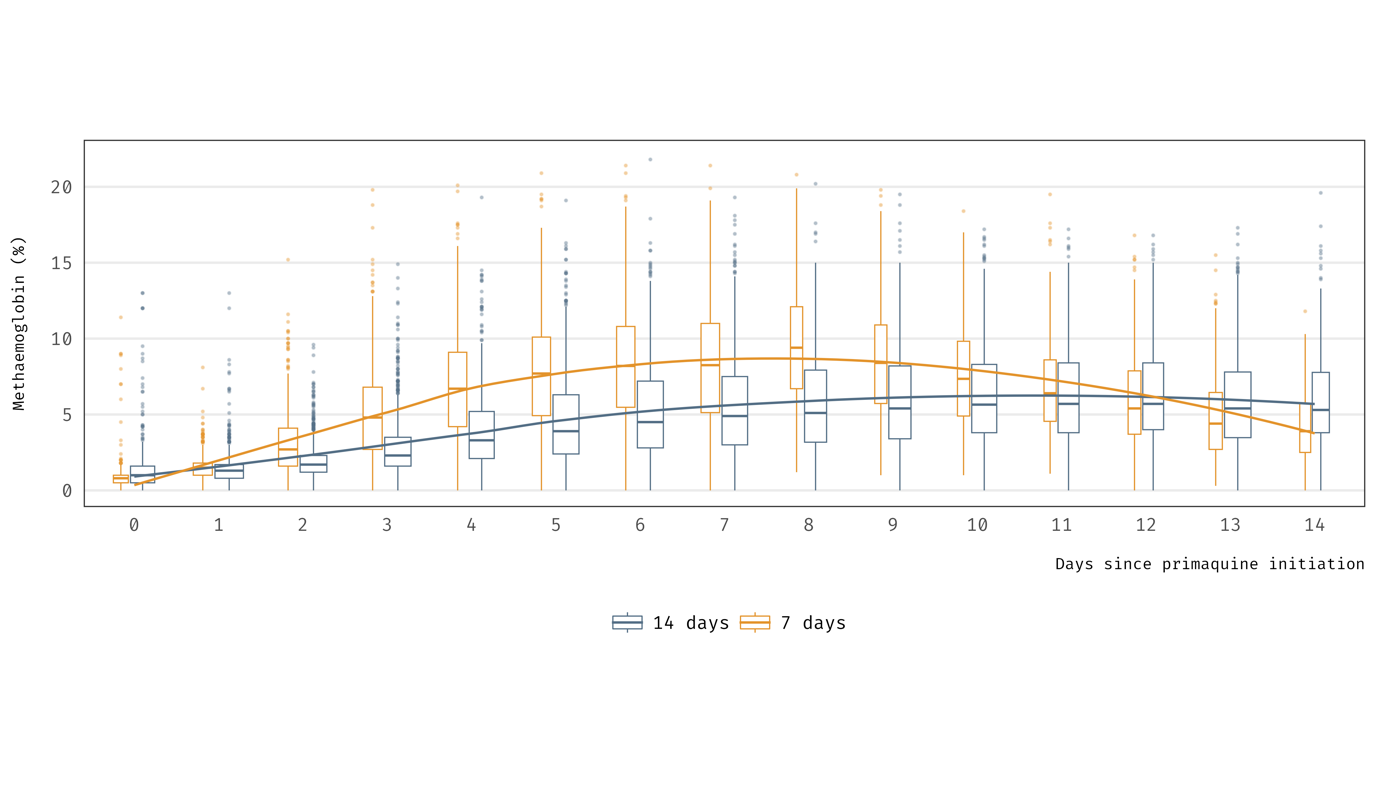
**

**Figure C. Dynamics of primaquine-induced increases in blood methaemoglobin over time**

Boxplot represents the distribution of methaemoglobin levels on a particular day following primaquine. Solid curve summarises the data points by primaquine regimen over time. Box width is proportional to the square root of the number of patients.


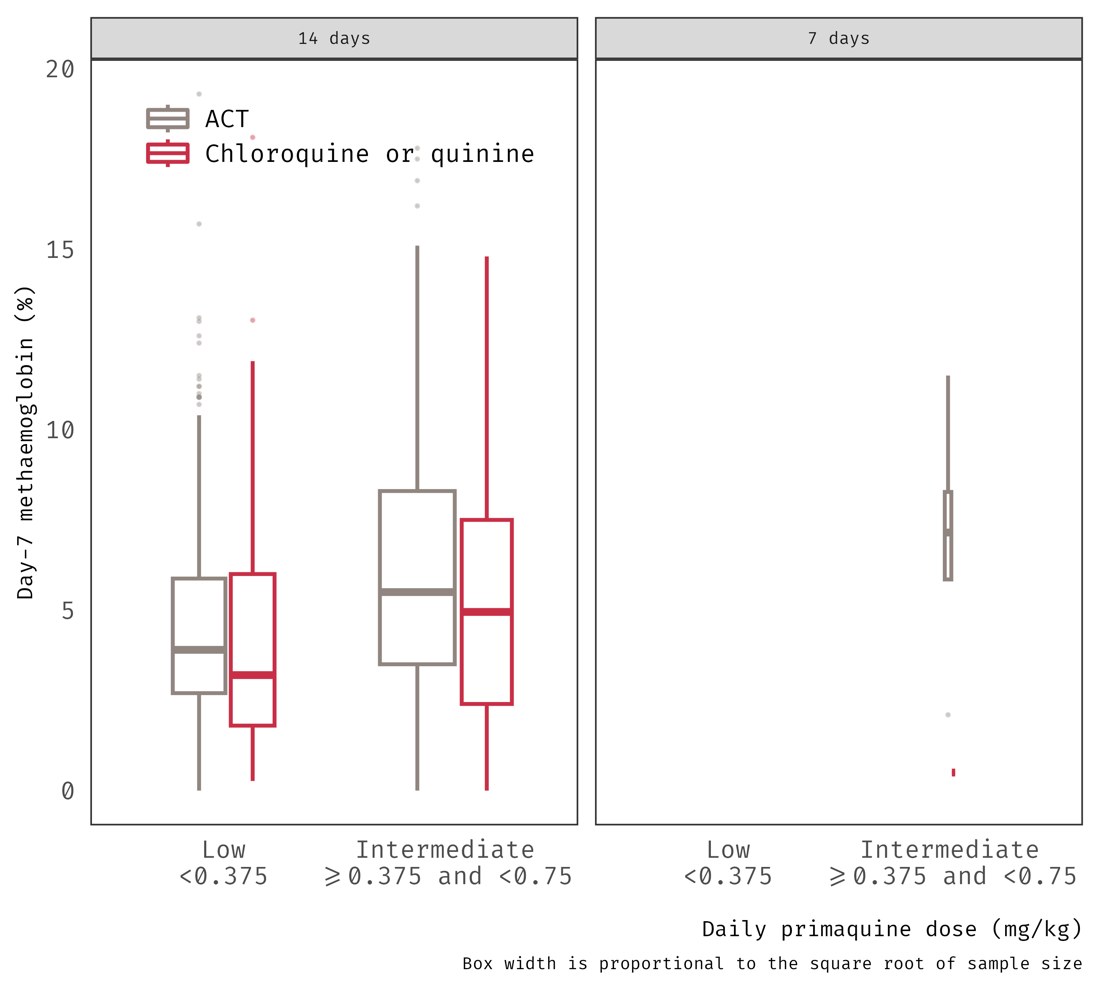


**Figure D. Day 7 methaemoglobin concentrations by primaquine regimen and dose group**

Among patients treated with a low-to-intermediate daily primaquine dose, day 7 methaemoglobin was lower when primaquine was combined with chloroquine or quinine as a partner drug. Most patients received a high daily primaquine dose in the 7-day regimen.

**
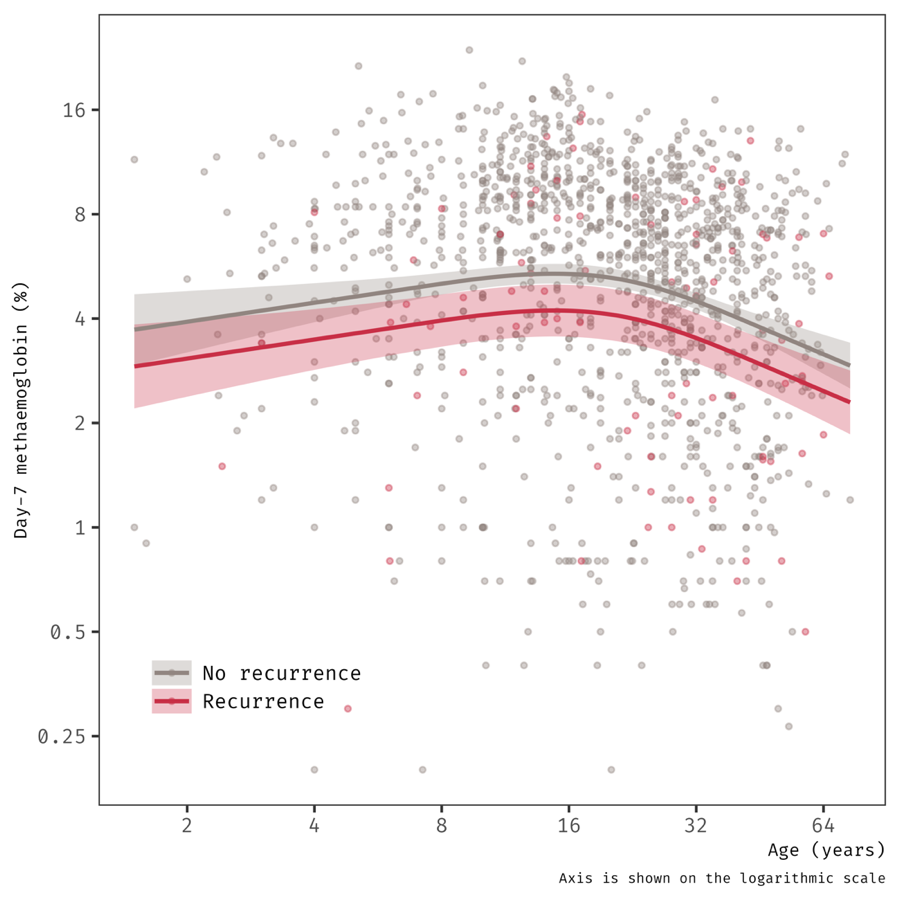
**

**Figure E. Inverse J-shaped association between patient age and day 7 methaemoglobin by recurrence status, after controlling for daily mg/kg primaquine dose**

Solid curve denotes a regression curve (with a 95% confidence interval) of day-7 methaemoglobin levels on age. Horizontal and vertical axes are shown on the logarithmic scale.


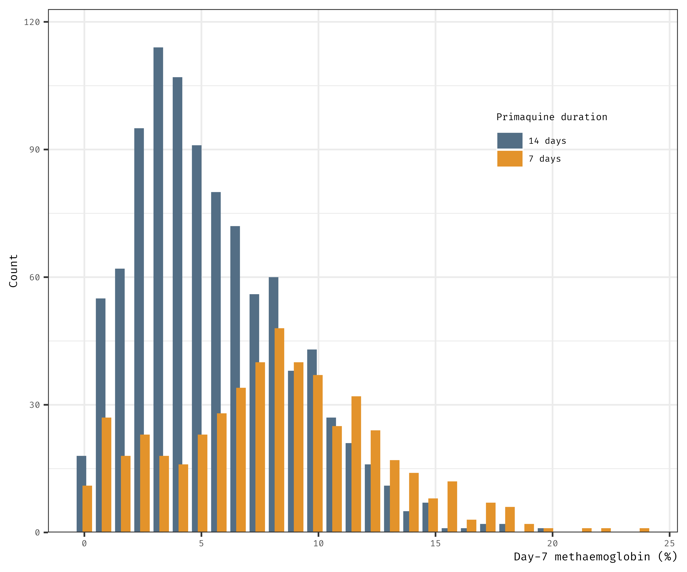

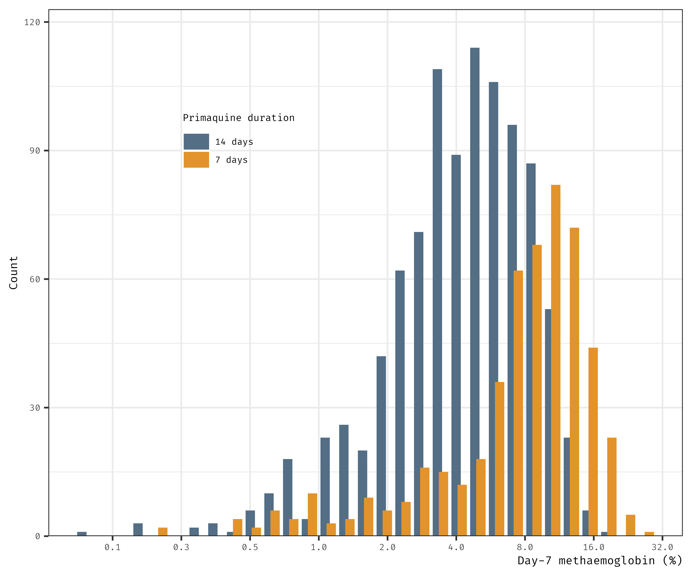


**B**

**A**

**Figure F. Distribution of day 7 methaemoglobin among the patients on (A) the original scale and (B) the logarithmic scale**


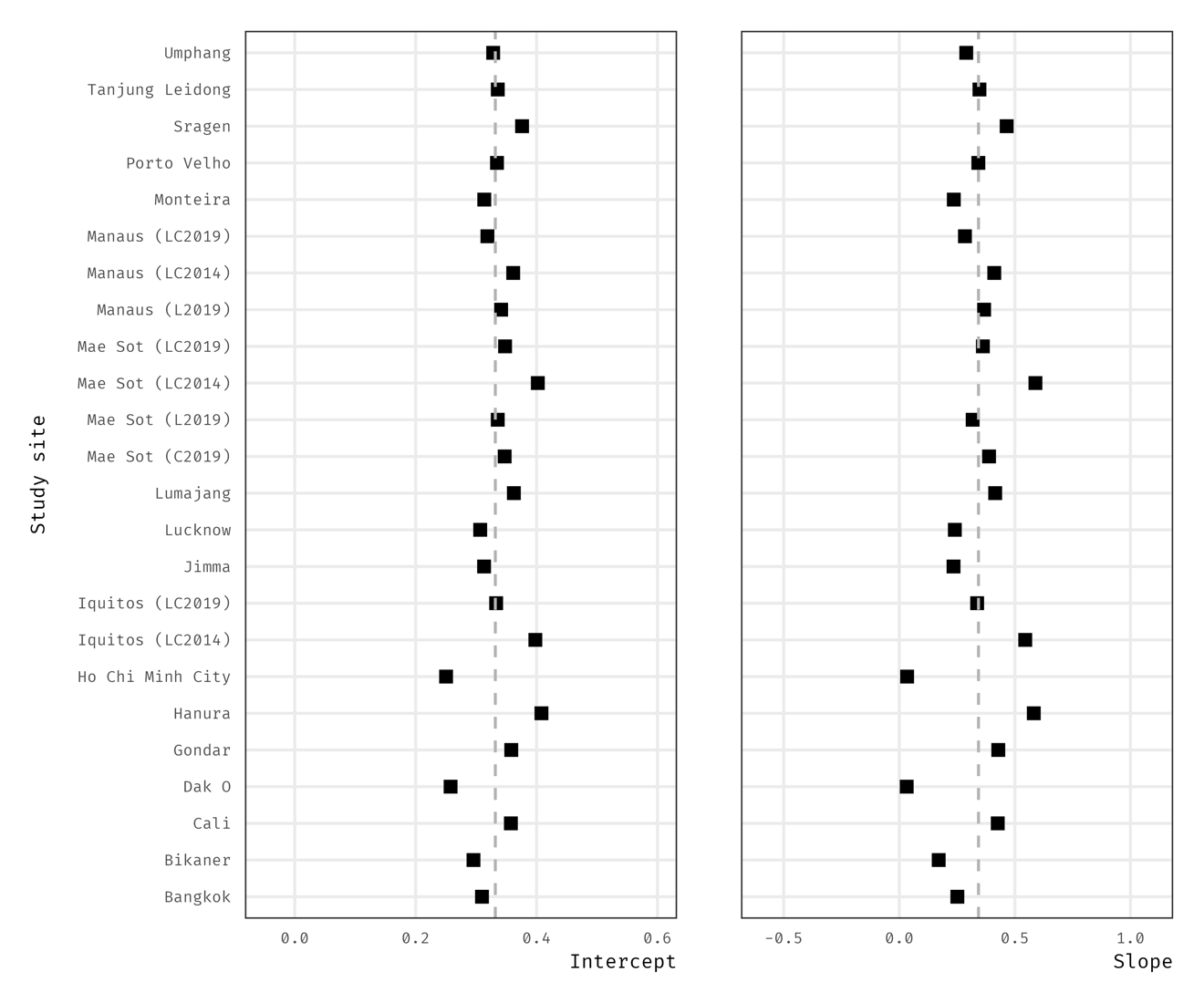


**Figure G. Mixed-effects estimates for the intercept and slope (mean difference)**

Slope represents the estimated mean difference of day-7 methaemoglobin levels for each additional 0.1 mg/kg/day increase in primaquine dose.

**Table F. Regression table output for the main Cox proportional hazards model**

|  | **Adjusted**  **hazard ratio** | **Standard error** | **Test**  **statistic** | **P value** |
| --- | --- | --- | --- | --- |
| **1. Log_2_(day-7 methaemoglobin levels)** | 0.698 | 0.103 | -3.49 | 0.0005 |
| **2. Weight-adjusted daily primaquine dose** | 2.66 | 2.87 | 0.341 | 0.7330 |
| **3. Primaquine duration regimen** | 1.16 | 0.779 | 0.188 | 0.8500 |
| **4. Within-site interaction between 2 & 3** | 0.0025 | 2.41 | -2.49 | 0.0129 |
| **4. Across-site interaction between 2 & 3** | 0.35 | 6.53 | -0.161 | 0.8720 |
| **5. Age** | 0.99 | 0.0082 | -1.17 | 0.2410 |
| **6. Sex** | 0.878 | 0.225 | -0.582 | 0.5610 |
| **7. Log_e_(*P. vivax* density)** | 1.28 | 0.0728 | 3.42 | 0.0006 |


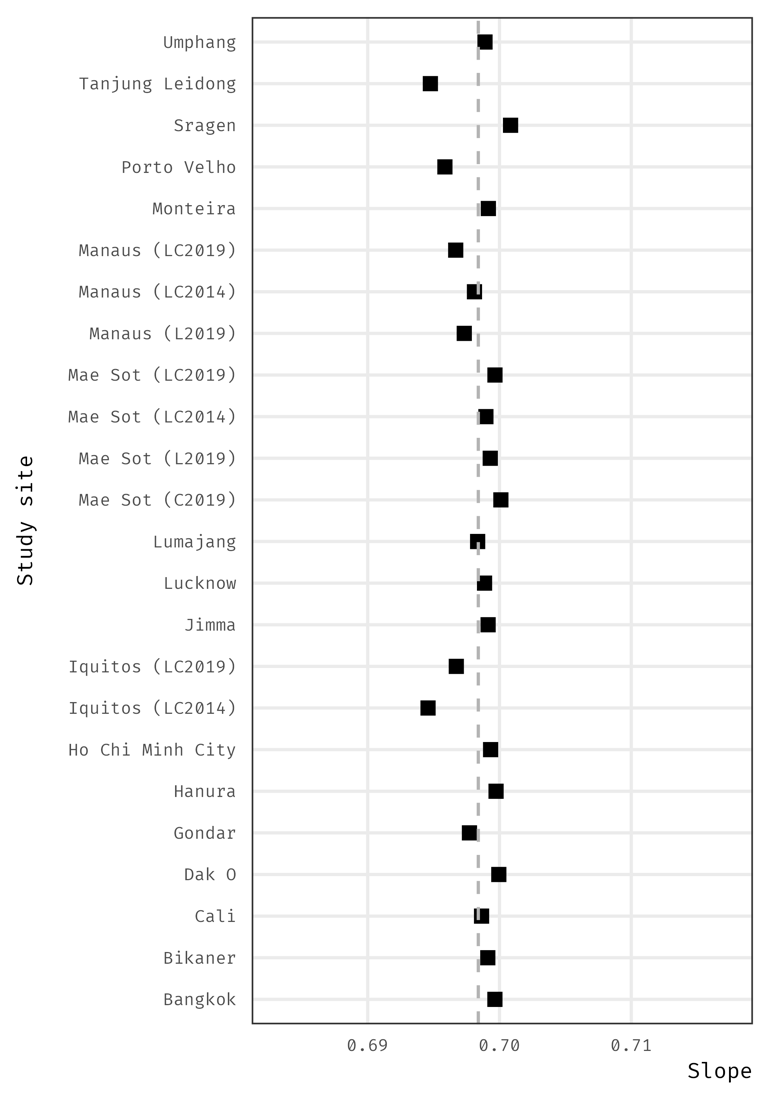


**Figure H. Mixed-effects estimates for the slope (hazard ratio)**

Slope represents the estimated adjusted hazard ratio of vivax recurrences for each doubling in day-7 methaemoglobin levels.

**
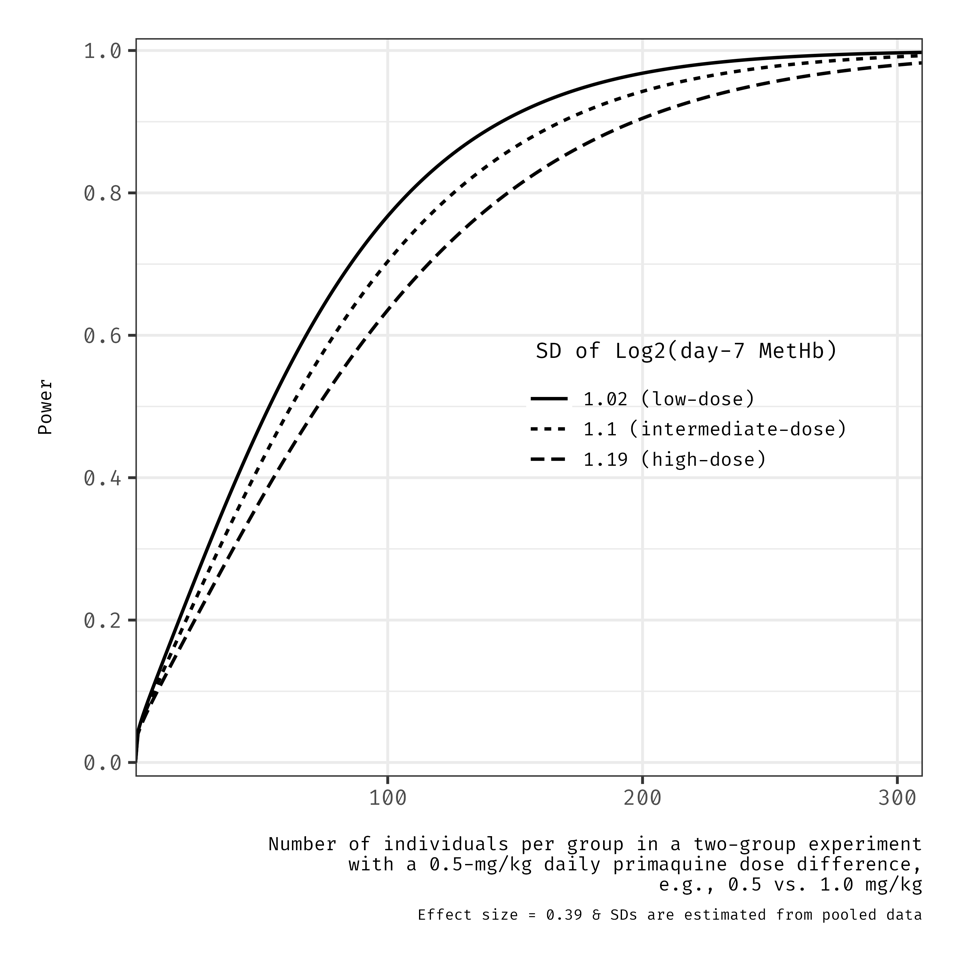
**

**Figure I. Example of sample size calculations for future studies**

The assumed effect size is 0.39 (change on the log_2_ day 7 methaemoglobin) which is equivalent to a 0.5-mg/kg increase in daily primaquine dose. The standard deviation (SD) of the log_2_ day 7 methaemoglobin level were calculated for different categories of daily mg/kg primaquine dose based on pooled data. The false positive rate was set to 5%. The population distribution of the log_2_ of day 7 methaemoglobin level conditional on the daily dose was assumed to follow a normal distribution.

 
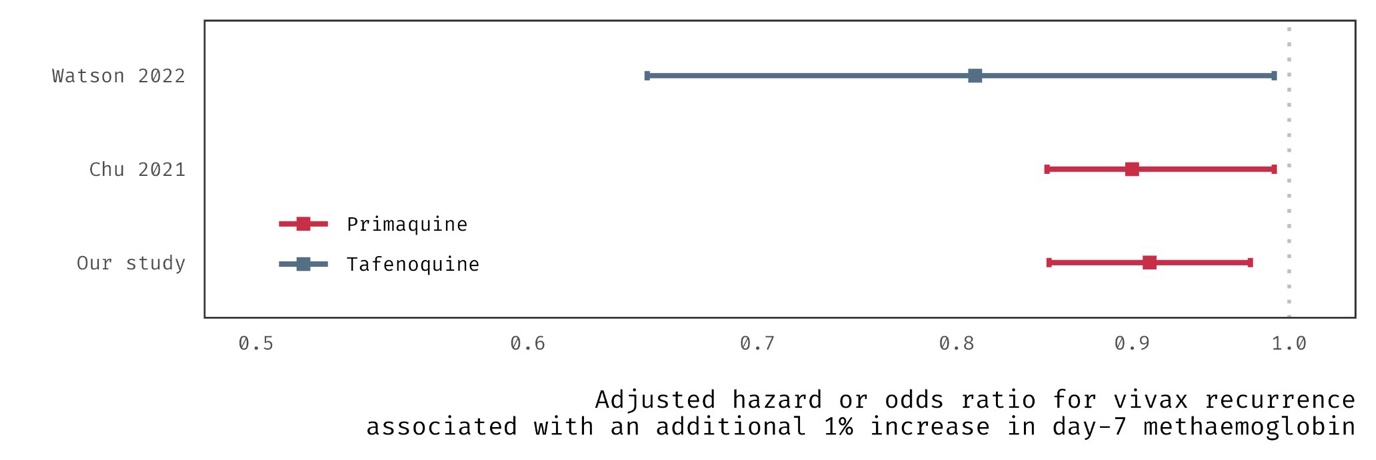


**Figure J. Comparisons of estimates from the current analysis with those previous studies**

Solid square represents a point estimate (with a 95% uncertainty interval). Horizontal axis is shown on the logarithmic scale.

References

1. Pasaribu AP, Chokejindachai W, Sirivichayakul C, Tanomsing N, Chavez I, Tjitra E, et al. A randomized comparison of dihydroartemisinin-piperaquine and artesunate-amodiaquine combined with primaquine for radical treatment of vivax malaria in Sumatera, Indonesia. J Infect Dis. 2013;208(11):1906-13. Epub 20130806. doi: 10.1093/infdis/jit407. PubMed PMID: 23926329; PubMed Central PMCID: PMCPMC3814843.

2. Sutanto I, Tjahjono B, Basri H, Taylor WR, Putri FA, Meilia RA, et al. Randomized, Open-Label Trial of Primaquine against Vivax Malaria Relapse in Indonesia. Antimicrob Agents Chemother. 2013;57(3):1128-35. doi: 10.1128/aac.01879-12.

3. Llanos-Cuentas A, Lacerda MV, Rueangweerayut R, Krudsood S, Gupta SK, Kochar SK, et al. Tafenoquine plus chloroquine for the treatment and relapse prevention of *Plasmodium vivax* malaria (DETECTIVE): a multicentre, double-blind, randomised, phase 2b dose-selection study. Lancet. 2014;383(9922):1049-58. Epub 20131219. doi: 10.1016/S0140-6736(13)62568-4. PubMed PMID: 24360369.

4. Nelwan EJ, Ekawati LL, Tjahjono B, Setiabudy R, Sutanto I, Chand K, et al. Randomized trial of primaquine hypnozoitocidal efficacy when administered with artemisinin-combined blood schizontocides for radical cure of *Plasmodium vivax* in Indonesia. BMC Med. 2015;13:294. Epub 20151211. doi: 10.1186/s12916-015-0535-9. PubMed PMID: 26654101; PubMed Central PMCID: PMCPMC4676167.

5. Chu CS, Phyo AP, Turner C, Win HH, Poe NP, Yotyingaphiram W, et al. Chloroquine Versus Dihydroartemisinin-Piperaquine With Standard High-dose Primaquine Given Either for 7 Days or 14 Days in *Plasmodium vivax* Malaria. Clin Infect Dis. 2019;68(8):1311-9. doi: 10.1093/cid/ciy735. PubMed PMID: 30952158; PubMed Central PMCID: PMCPMC6452005.

6. Lacerda MVG, Llanos-Cuentas A, Krudsood S, Lon C, Saunders DL, Mohammed R, et al. Single-Dose Tafenoquine to Prevent Relapse of *Plasmodium vivax* Malaria. N Engl J Med. 2019;380(3):215-28. doi: 10.1056/NEJMoa1710775. PubMed PMID: 30650322; PubMed Central PMCID: PMCPMC6657226.

7. Llanos-Cuentas A, Lacerda MVG, Hien TT, Vélez ID, Namaik-Larp C, Chu CS, et al. Tafenoquine versus Primaquine to Prevent Relapse of *Plasmodium vivax* Malaria. N Engl J Med. 2019;380(3):229-41. doi: 10.1056/NEJMoa1802537. PubMed PMID: 30650326; PubMed Central PMCID: PMCPMC6657225.

8. Taylor WRJ, Thriemer K, Von Seidlein L, Yuentrakul P, Assawariyathipat T, Assefa A, et al. Short-course primaquine for the radical cure of *Plasmodium vivax* malaria: a multicentre, randomised, placebo-controlled non-inferiority trial. Lancet. 2019;394(10202):929-38. doi: 10.1016/s0140-6736(19)31285-1.

9. Becker RAW, A. R.; Brownrigg, R.; Minka, T. P.; Deckmyn, A. maps: Draw Geographical Maps. 3.4.1 ed: R package; 2023.

10. Wickham HC, W.; Henry, L.; Pedersen, T. L.; Takahashi, K.; Wilke, C.; Woo, K.; Yutani, H. ggplot2: Create Elegant Data Visualisations Using the Grammar of Graphics. 3.5.1 ed: R package; 2023.

11. Battle KE, Karhunen MS, Bhatt S, Gething PW, Howes RE, Golding N, et al. Geographical variation in *Plasmodium vivax* relapse. Malar J. 2014;13(1):144. doi: 10.1186/1475-2875-13-144.

12. Battle KE, Lucas TCD, Nguyen M, Howes RE, Nandi AK, Twohig KA, et al. Mapping the global endemicity and clinical burden of *Plasmodium vivax*, 2000–17: a spatial and temporal modelling study. Lancet. 2019;394(10195):332-43. doi: 10.1016/s0140-6736(19)31096-7.

13. Solari-Soto L, Soto-Tarazona A, Mendoza-Requena D, Llanos-Cuentas A. Ensayo clínico del tratamiento de la malaria vivax con esquema acortado de primaquina comparado con el esquema tradicional. Rev Soc Peru Med Interna. 2002;15:196-9.

14. Carmona-Fonseca J. Vivax malaria in children: recurrences with standard total dose of primaquine administered in 3 vs. 7 days. Iatreia. 2010;23(1):10-20.

15. Ley B, Alam MS, Thriemer K, Hossain MS, Kibria MG, Auburn S, et al. G6PD deficiency and antimalarial efficacy for uncomplicated malaria in Bangladesh: a prospective observational study. PloS One. 2016;11(4):e0154015.

16. Fukuda MM, Krudsood S, Mohamed K, Green JA, Warrasak S, Noedl H, et al. A randomized, double-blind, active-control trial to evaluate the efficacy and safety of a three day course of tafenoquine monotherapy for the treatment of *Plasmodium vivax* malaria. PLoS One. 2017;12(11):e0187376.

17. Chu CS, Phyo AP, Lwin KM, Win HH, San T, Aung AA, et al. Comparison of the cumulative efficacy and safety of chloroquine, artesunate, and chloroquine-primaquine in *Plasmodium vivax* malaria. Clin Infect Dis. 2018;67(10):1543-9.

18. Moore BR, Salman S, Tobe R, Benjamin J, Yadi G, Kasian B, et al. Short-course, high-dose primaquine regimens for the treatment of liver-stage vivax malaria in children. Int J Infect Dis. 2023;134:114-22.

19. Sutanto I, Soebandrio A, Ekawati LL, Chand K, Noviyanti R, Satyagraha AW, et al. Tafenoquine co-administered with dihydroartemisinin–piperaquine for the radical cure of *Plasmodium vivax* malaria (INSPECTOR): a randomised, placebo-controlled, efficacy and safety study. Lancet Infect Dis. 2023.
