# Supplementary material for: Methaemoglobin as a surrogate marker of primaquine antihypnozoite activity in Plasmodium vivax malaria: A systematic review and individual patient data meta-analysis
Source: PLoS Med. 2024 Sep 27;21(9):e1004411. doi: 10.1371/journal.pmed.1004411 (PMC11469483; doi:10.1371/journal.pmed.1004411)
Supplement: Attachment — Submitted filename: fadilah.pdf [file pmed.1004411.s002.pdf]

This paper presents a systematic review with individual participant meta–analysis of studies developing a surrogate marker in *P vivax* infection.

I was asked for a methodological report and I interpret that to include all aspects of the design and conduct of the study.

Points of detail

Page 1 When I read a study involving transmission of data–sets between centres I always feel more confident if the donor sites appear in the affiliations. This sort of transmission is both tedious and open to error. In this case some of the sites do not seem to appear. Is this because some contributing studies were multi–country and so the curation of data happened within study?

Page 5 The two sentences from 'If the day 7' seem a rather convoluted way of saying that since the physiological level is 1 zeroes were replaced with 1 before log–transforming.

Page 5 When I first read this I came up short on reading 'the estimated predictive effect of each doubling' which made me look back and realise I had missed the subscript in $\log_2$. For the benefit of other careless readers perhaps either move that section of text up to after the explanation of the transformation or spell out base 2 on the first occurrence.

Page 6 Indeed this is precisely the scenario where Riley et al. (2023) recommend the one–stage approach although they are fairly neutral otherwise.

Page 6 The forest plot is for study rather than site which I think needs explaining. I know from looking at the code that the authors seem aware of the lack of independence between some of the estimates but it could be remarked on here too.

Page 6 If the analysis used any packages above what comes as a standard installation it would be good to cite them. For instance I see in the code that metafor was used. I do not think we need citation for packages purely involved in data management and graphing. (Disclaimer: I am a CRAN package author.)

Page 9 The proposal about sample size determination is important but I think it could use some expansion here or possibly on page 6 in the methods. At present it is very compact

Page 9 I see in the code that the authors created a table of the other researchers' estimates quoted here. That might be informative (and have more impact) here.

Page 10 The sentence about skin pigment follows a section on possible improvements to measurement but it reads the other way. Surely what is needed is more accurate not less accurate.

Figure S5 I am all in favour of showing all the raw data in graphs but this is not a great success. Is there some other plot which could be shown? Figure 4 on page 22 is, for some reason, clearer.

Points of more substance

The authors state on page 8 that estimates were consistent across sites. I think a display of these estimates would be helpful. If I understand it correctly the primary studies did not use identical protocols and so there might be expected to be some clinical heterogeneity. Our confidence in the results would be enhanced if we could see that any resulting statistical heterogeneity was small. Since the data is rather sparse conventional heterogeneity measures like I^2 may not work but presumably we could see the estimated random intercepts and slopes perhaps even plotted one against the other. We do need some formal statement about this since, sadly, these days nobody trusts scientists and their eyeballing of the data.

Summary

Mostly for clarifications. I do think the point about heterogeneity is important but I am agnostic about ways of presenting it.

Michael Dewey

References

R D Riley, J Ensor, M Hattle, K Papadimitriopoulou, and T P Morris. Two–stage or not two–stage? That is the question for IPD meta–analysis projects. *Research Synthesis Methods*, 14:903–910, 2023.
