# Supplementary material for: Methaemoglobin as a surrogate marker of primaquine antihypnozoite activity in Plasmodium vivax malaria: A systematic review and individual patient data meta-analysis
Source: PLoS Med. 2024 Sep 27;21(9):e1004411. doi: 10.1371/journal.pmed.1004411 (PMC11469483; doi:10.1371/journal.pmed.1004411)
Supplement: Attachment — Submitted filename: Responses to reviewers.docx [file pmed.1004411.s003.docx]

Editor

PLOS Medicine

9 June 2024

Dear Editor,

We are grateful to the reviewers and editors for their helpful comments.

We have attached a revised manuscript "**Methaemoglobin as a surrogate marker of primaquine antihypnozoite activity in *Plasmodium vivax* malaria: a systematic review and individual patient data meta-analysis**" (PMEDICINE-D-24-01457R1).

Please also find the point-by-point responses to the comments below.

Thank you for your consideration.

Yours sincerely,

On behalf of authors,

Ihsan Fadilah, MD MSc

James A. Watson, DPhil

**Reviewer #1**

Comment #1

Timely conducted research project. The relapses of P vivax is one of the problem for elimination of malaria from countries. This has given an insight how to predict relapses. However, this needs to conduct in field level to see the practicability of measuring methemoglobin in low resource settings. Further, need to conduct future studies to determine dose and regime of primaquine/tafenoquine to obtain maximum level of methemoglobin.

Author response

We thank the reviewer for their positive comments and agree that predicting relapses at the individual patient level in clinical practice is potentially important. However, we do not believe that methaemoglobin is a substitute for clinical endpoints, but rather a useful adjunctive measure. A key confounder on the utility of methaemoglobin, is the substantial inter-individual variability in hypnozoite burdens, that impact the pretreatment probability of relapse independent of day 7 methaemoglobin. Hence, our study supports the use of day 7 methaemoglobin at the population level when comparing primaquine regimens or doses. This could even be used in healthy volunteer studies. As Reviewer #1 indicates, monitoring methaemoglobin can provide important information on whether the optimal dose and regimen of primaquine or tafenoquine have been achieved.

**Reviewer #2**

Comment #1

**Page 1** When I read a study involving transmission of data–sets between centres I always feel more confident if the donor sites appear in the affiliations. This sort of transmission is both tedious and open to error. In this case some of the sites do not seem to appear. Is this because

some contributing studies were multi–country and so the curation of data happened within study?

Author response

Shared data were uploaded to the Worldwide Antimalarial Resistance Network (WWARN) data repository for curation and standardisation. WWARN is a well-established data platform for data re-use. All studies are curated to a single data storage format using SDTM, and this process involves extensive checking of the individual datasets by trained data engineers. This process has underpinned many large individual patient data meta-analyses, and most of the data used for this study were already curated in the repository. WWARN uses a collaborative study group model, whereby all data contributors are asked to participate and be co-authors in published outputs. For each study included in this meta-analysis, the original study PIs are authors on the paper and have checked the results. However, as the reviewer states, some studies were multi-country, and thus data were curated within the study and not every single site is listed in the affiliations.

Comment #2

**Page 5** The two sentences from ‘If the day 7’ seem a rather convoluted way of saying that since the physiological level is 1 zeroes were replaced with 1 before log–transforming.

Author response

We agree that this was unclear and we have revised the sentences (lines 192­–195) to make this clearer:

"If the day 7 methaemoglobin percentage was recorded as 0, we replaced it with 1, assuming an approximately physiological methaemoglobin level of 1%­­. Zero recordings are likely to represent mis-readings of the analytical machine."

Comment #3

**Page 5** When I first read this I came up short on reading ‘the estimated predictive effect of each doubling’ which made me look back and realise I had missed the subscript in log2. For the benefit of other careless readers perhaps either move that section of text up to after the explanation of the transformation or spell out base 2 on the first occurrence.

Author response

We have now used 'base 2' (lines 191–192) to replace the previous subscript as follows.

"The day 7 methaemoglobin percentage (log base 2 transformed) was included in the statistical model as a continuous variable."

Comment #4

**Page 6** Indeed this is precisely the scenario where Riley et al. (2023) recommend the one–stage approach although they are fairly neutral otherwise.

R D Riley, J Ensor, M Hattle, K Papadimitriopoulou, and T P Morris. Two–stage or not two–stage? That is the question for IPD meta–analysis projects. Research Synthesis Methods, 14:903–910, 2023.

Author response

We thank Reviewer #2 for pointing out this relevant paper. We have now added this to the reference list.

Comment #5

**Page 6** The forest plot is for study rather than site which I think needs explaining. I know from looking at the code that the authors seem aware of the lack of independence between some of the estimates but it could be remarked on here too.

Author response

We have now added clarification (lines 231–233) that, because the primaquine regimen was deemed the primary determinant of relapse risk, the correlation in the estimates from the same study could be mostly accounted for.

"Assuming that the primaquine regimen was the primary determinant of variation in the outcome, some dependence in the estimates derived from the same study [9, 21] was expected to be minimal."

Comment #6

**Page 6** If the analysis used any packages above what comes as a standard installation it would be good to cite them. For instance I see in the code that metafor was used. I do not think we need citation for packages purely involved in data management and graphing. (Disclaimer: I am a CRAN package author.)

Author response

We have now included citations for the main R packages used for modelling (lines 272–273).

"Statistical modelling was implemented using the R packages survival [25], coxme [26], metafor [27], rms [28], and lme4 [29]."

Comment #7

**Page 9** The proposal about sample size determination is important but I think it could use some expansion here or possibly on page 6 in the methods. At present it is very compact.

Author response

We have expanded the Methods section on the sample size determination (lines 257–267).

We have added:

“We estimated the standard deviation of the log_2_ day 7 methaemoglobin level, conditional on the daily mg/kg primaquine dose from the pooled data. For example, the standard deviation for the high daily dose group is approximately 1.19. Assuming a normal distribution for the log_2_ day 7 methaemoglobin conditional on the daily dose allows for a simple calculation of the required sample size. This is derived from a t-test for a difference between two normal distributions with mean difference (effect size) of 0.39 and equal standard deviations in both groups. For the power calculation for the clinical endpoints, we assumed recurrence risks of 16% versus 8% (corresponding to primaquine doses of 0.5 versus 1 mg/kg over 7 days) [23].”

Comment #8

**Page 9** I see in the code that the authors created a table of the other researchers’ estimates quoted here. That might be informative (and have more impact) here.

Author response

We have now included a graphical output for comparing the estimates in Supporting Information.

Lines 403–405:

"These estimates suggested that an increase of one percentage-point of day 7 methaemoglobinaemia was associated with an estimated reduction of 10% for primaquine and 20% for tafenoquine in the risk of *P. vivax* recurrence (Fig S10 provides a graphical comparison)."

Comment #9

**Page 10** The sentence about skin pigment follows a section on possible improvements to measurement but it reads the other way. Surely what is needed is more accurate not less accurate.

Author response

We have moved the sentence about skin pigment to immediately follow the other limitations regarding methaemoglobinaemia as an inherently noisy measure (lines 454–458).

"It is important to note that methaemoglobinaemia itself is an inherently noisy measure with substantial variation between patients, especially in patients receiving higher doses of 8-aminoquinoline treatment (Fig S7 shows higher methaemoglobin variability in higher dose groups). There is also a possibility of less accurate methaemoglobin measurements by using CO-oximetry for patients with darker skin [46]. ..."

Comment #10

**Figure S5** I am all in favour of showing all the raw data in graphs but this is not a great success. Is there some other plot which could be shown? Figure 4 on page 22 is, for some reason, clearer.

Author response

Figure S5 shows the relationship between age and day 7 methamoglobin, both on the log scale. Although we agree that the figure is a bit difficult to read, this is a consequence of the wide inter-individual variability in day 7 methaemoglobin responses. Figure 4 shows the same data but with primaquine daily dose on the x-axis. We believe that presentation of the raw data provides important information for the reader on the spread of individual measurements and would like to retain the figure as it stands.

Comment #11

The authors state on page 8 that estimates were consistent across sites. I think a display of these estimates would be helpful. If I understand it correctly the primary studies did not use identical protocols and so there might be expected to be some clinical heterogeneity. Our confidence in the results would be enhanced if we could see that any resulting statistical heterogeneity was small. Since the data is rather sparse conventional heterogeneity measures like *I^2^* may not work but presumably we could see the estimated random intercepts and slopes perhaps even plotted one against the other. We do need some formal statement about this since, sadly, these days nobody trusts scientists and their eyeballing of the data.

Author response

Although it is correct that the clinical protocols differed between studies, the main differences that matter for this analysis are the site level relapse proportions (this is a function of the malaria transmission intensity), the doses of primaquine used (all studies used once daily dosing regimens), the duration of treatment with primaquine, and the exact timing of the follow-up methaemoglobin measurements. In addition, there may be genetic factors driving methaemoglobin response that related to ethnicity that differ across countries and regions.

We used between-site standard deviation to describe heterogeneity across the estimates. To further support the statements, we have now added two plots that show the mixed-effects estimates across the study sites in Supporting Information. However, we also acknowledge that interpreting the extent of heterogeneity can be subjective and depends on the context.

Lines 336 and 353:

"... Fig S7 shows the study-site specific estimates..."

"... Fig S8 shows the study-site specific estimates..."

**Reviewer #3**

Comment #1

The authors have conducted systematic review and pooled analysis of individual participant data from 8 clinical trials across 9 geographical sites to study the association between day 7 met-hemoglobin levels and the sub-sequent risk of recurrent parasitemia in individuals treated with primaquine (an 8-aminoquinoline) for radical cure of P. vivax hypnozoites.

The paper is very well written and methodologically sound. It provides important insights regarding day 7 met-hemoglobin as a surrogate marker for treatment outcome as well as insights regarding the potential mechanisms by which 8-aminoquinolines act on dormant liver-stage parasites. The findings could have impact on the design future studies on drug efficacy.

The findings are of high interest for researchers studying the effect 8-aminoquinolines and related compounds as well as for researchers conducting clinical trials on radical cure of dormant plasmodium liver-stage parasites and/or sexual stages.

My main concern is that although day 7 met-hemoglobin appears promising as a surrogate marker of treatment outcome on average, there appears to be a substantial overlap in levels between individuals who suffer parasite recurrence and individuals who do not. To what extent 7 meth-hemoglobin levels can accurately predict treatment outcome on the individual level remains unclear from the results presented in the manuscript. I believe this is important to assess in order to evaluate the usefulness of day 7 meth-hemoglobin as a surrogate marker of treatment outcome. I would therefore like to ask the authors to complement the results with an analysis of the ability of day 7 meth-hemoglobin to predict the odds of recurrence (or time to recurrence) on the individual level.

Should the authors believe that this is not relevant, I believe this has to be explained and motivated clearly throughout the manuscript.

Author response

We thank the reviewer for their insightful comments.

We agree that there is substantial inter-individual variation in methaemoglobin response to primaquine treatment. In addition, there will also be substantial inter-individual heterogeneity in the risk of relapse (i.e., heterogeneity in the number of liver hypnozoites). Taken together, we believe this precludes the use of methaemoglobin as an individual level predictor. We acknowledge that this key point was not clear in our original Introduction, Results, and Discussion. We have therefore revised the text to emphasise this point.

The goal of our study is to validate the use of methaemoglobin as a surrogate marker of anti-hypnozoite effect at the population-level.

In the Introduction, we have changed the last sentence to:

“We examined the utility of methaemoglobin as **a population-level surrogate endpoint** for vivax recurrence in a pooled individual patient data meta-analysis using all available data from *P. vivax* patients treated with primaquine.”

In the Discussion (lines 490–501), we have added:

“The goal of this analysis was not to establish day-7 methaemoglobin levels as an individual level predictor of relapse risk. Predicting relapse at the individual level is very difficult because there is large inter-individual variation in liver hypnozoite burdens [1, 49]. A patient may have no methaemoglobin response to primaquine and not subsequently relapse because they had no hypnozoites at enrolment. Indeed, in no primaquine treated arms of the included studies, the risk of any recurrent illness by day 120 ranged from around 25% to 80%.”

Comment #3

Apart from that I only have a few very minor suggestions:

Lines 436 - 449: In this paragraph the authors discuss variability in measurement of methhemoglobin particularly related to transcutaneaous measurement. It could be worth commenting whether or not if "invasive" measurement through analysis of capillary blood on e.g. on a blood-gas analyzer (also CO-oximetry based) could reduce measurement variability.

Author response

We thank the reviewer for the input and have added this to our manuscript (lines 461–463).

"More invasive methods, such as directly measuring the biomarker in capillary blood, could enhance measurement accuracy but are less acceptable and would be logistically challenging in low resource settings."

Comment #4

Figure legends - This is a general comment but figure legends do not specify what the trend lines in the different figures represent. Please clarify this to avoid any confusion.

Author response

We have revised the text for the figure legends to provide additional descriptions of the plots with lines or curves throughout the manuscript.

Comment #5

Finally, I think it would be valuable if the authors included the regression table (at least for the main analysis based on the cox proportional hazards model) as supplementary information for the interested reader to scrutinize.

Author response

We have added a regression table for the main Cox model for more detail in Supporting Information.

Line 349:

"... Table S6 shows an excerpt of the regression output..."

**Reviewer #4**

Comment #1

This article by Fadilah et al. describes a comprehensive review of literature on clinical trials that explored primaquine use to clear hypnozoite (dormant) forms of P. vivax. Beginning with 219 trials, the authors used well thought out criteria to narrow their analysis to 8 trials, containing 1,747 G6PD-normal patients spread across 8 countries. The goal was to compare day 7 methemoglobinemia levels with time to relapse. Their analysis showed that a doubling on day 7 of methemoglobin percentage was associated with an estimated 30% reduction in the risk of vivax recurrence following primaquine administration. The authors provide a compelling argument that measuring the methemoglobin levels on day 7 is far preferable, logistically and financially, to extending studies and assessing whether recrudescence occurred (with those trials typically extending up to 120 days). This makes logical sense. I should also note that many of the world leading malaria physicians and modelers working on malaria in SE Asia are on this manuscript. Overall, I found this to be an excellent study. It would have been clearly state textually how many trials would have enabled an analysis of tafenoquine.

Early and cost-effective screening that would predict recrudescence in P. vivax-infected patients is an important topic. The authors have put together an excellent analysis. While this is a systematic review of trials data, and not a new trial itself, I find the topic and the rigor of the analysis a good fit for PLoS Medicine. The findings merit publication in this high-level journal. Although rare for me, I found nothing that required revision.

Author response

We thank the reviewer for their positive comments and support for publication in PLOS Medicine.

**Reviewer #5**

Comment #1

Summary: In this manuscript, the authors present a systematic data analysis of clinical trials involving the treatment with primaquine of P. vivax infected patients with the goal to validate methaemoglobin as a surrogate marker of anti-relapse efficacy of this 8-aminoquinoline drug. The authors applied a stringent selection of data from 8 studies out of 219 representing 1747 patients (out of 4122) in 24 study sites and 8 countries. The analysis, carefully conducted by the authors, shows that an increase of the primaquine dose correlates with an increase of methaemoglobin. A 2-fold increase of methaemoglobin percentage in blood of patients is associated with a 30% decrease in the risk to see P. vivax relapsing in such patients, thus validating methaemoglobin as a surrogate marker of the anti-relapse efficacy of primaquine.

The study is well conducted and clearly reported in this manuscript. The method is well described, and figures are self-explanatory.

I am very supportive of the publication of this manuscript in PLoS Medicine, provided that the authors adequately address the points raised below.

Major comments:

1) The authors should explain the statistical method they used to validate methaemoglobin as a surrogate of anti-relapse efficacy prognostic for primaquine after a stringent selection that consisted of keeping only 8 out of 219 clinical trials considered. The number of patients remaining in the analysis is certainly high enough to achieve the statistical power; however, it would be good to develop that aspect to further strengthen the manuscript.

Author response

We thank the reviewer for their comments. We note that there were 219 trials identified from the living systematic review of vivax malaria studies. However, most of these trials did not administer primaquine treatment or did not measure methaemoglobin, i.e., they provide no relevant information for our analysis. Only 13 studies provided relevant information, of which two did not have methaemoglobin measurements in the correct timeframe. Only three relevant studies were omitted from our analysis because we were unable to obtain individual level data. Hence, we obtained IPD data for 8/11 studies. Four of these studies were multi-country studies. Hence our analysis includes all the largest clinical studies done in recent years in vivax malaria and represents a spectrum of transmission settings supporting the generalisability our findings.

We added to the main text of the results:

“After review, 206 studies were excluded (almost all because they did not measure methaemoglobin, or did not have a primaquine arm which started in the first 3 days of treatment), leaving 13 studies eligible for the pooled analysis.”

Comment #3

2) Figure 1 is interesting but, in my opinion, should be the last one in the manuscript, supporting the discussion and integrating all the points discussed with CYP450-dependent metabolism of primaquine, possible absence of sporozoites yet detection of methaemoglobin, G6PD deficiency and absence of methaemoglobin (despite the fact that G6PD deficient patients are not considered in this study), etc… to provide readers with a more integrated view of the regulations of parasitemia and drug level of the metabolic intermediate in the liver associated to methaemoglobin.

Author response

We will defer to the editorial opinion on this comment. We would prefer to keep Figure 1 in first place because it clearly displays the assumed theoretical structural model underpinning the use of methaemoglobin as a surrogate outcome.

Comment #4

Minor comments:

Lines 292: Figure 2 shows weight-adjusted… should read Figure S2.

Line 318: Figure 6 is mentioned but here again Figure S6 is the valid one.

Line 425 : …a day 7 methaemoglobin should be amended as follow: … a day 7 methaemoglobin measurement…

Legend of figure 4 last sentence: "Vertical axis is show on the logarithmic scale" should read: "Vertical axis is shown on the logarithmic scale.

Author response

We have corrected the errors as suggested.

**Editorial Team**

Comment #1

The editorial team feels that the point raised by reviewer #3 regarding the "substantial overlap in levels between individuals who suffer parasite recurrence and individuals who do not" is very important - please address this comment carefully and consider adding an analysis of the ability of day 7 methemoglobin to predict the odds of recurrence (or time to recurrence) at the individual level.

We also ask that you further discuss clinical utility in the context of standard biomarker performance metrics.

Author response

As described under the Reviewer #3 section, we have now revised the text to clarify the distinction between our study's scope and establishing a clinical predictor for individual risk outcomes. We believe that presenting standard predictive performance metrics such as specificity, sensitivity, and positive and negative predictive values would not be relevant in our context.

As depicted in Figure 4, the overlapping levels between individuals who did and did not develop recurrences were shown in a "crude" manner for illustrative purposes only. Our conclusions are based on the multivariable Cox model, which objectively estimates the different levels between groups. Given the multidimensional nature of these results, which are not easily represented graphically, we presented the main findings numerically
