# Supplementary material for: Methaemoglobin as a surrogate marker of primaquine antihypnozoite activity in Plasmodium vivax malaria: A systematic review and individual patient data meta-analysis
Source: PLoS Med. 2024 Sep 27;21(9):e1004411. doi: 10.1371/journal.pmed.1004411 (PMC11469483; doi:10.1371/journal.pmed.1004411)
Supplement: Attachment — Submitted filename: Responses to reviewers (round 2).docx [file pmed.1004411.s004.docx]

Editor

PLOS Medicine

13 August 2024

Dear Editor,

We are grateful to the reviewers and editors for their additional comments.

We have attached a revised manuscript "**Methaemoglobin as a surrogate marker of primaquine antihypnozoite activity in *Plasmodium vivax* malaria: a systematic review and individual patient data meta-analysis**" (PMEDICINE-D-24-01457R1).

Please also find the point-by-point responses to the comments below.

Thank you for your consideration.

Yours sincerely,

On behalf of authors,

Ihsan Fadilah, MD MSc

James A. Watson, DPhil

**Editors**

Comment #1

We noticed that you stated in the manuscript that you "included randomized therapeutic trials and prospective cohort studies published between January 1, 2000 and September 29, 2022". We are not sure if the above statement means that your last search was conducted in 2022, or if no studies were eligible after September 2022. Please note that we normally expect searches to be updated within 6 months of submission. However, we recognize that providing an update for individual patient data can be a significant undertaking. Therefore, we will not make this an editorial requirement in your case. To provide more transparency, we suggest that you perform an updated search and include in the limitations sections of the discussion how many potential studies could be included with an updated search.

We had limited our systematic search to 29 September 2022 due to the considerable delays in accessing individual patient data and completing the analysis, but acknowledge that any more recent publications will not have been included.

To address this we have updated our search to identify studies up to 26 July 2024. Two additional studies were eligible, although it is unclear whether the authors would have agreed to share the data.

We have now added the characteristics of these studies to Supporting Information and included in the main text:

"We conducted a systematic search of Medline, Embase, Web of Science, and the Cochrane Library based on an existing living systematic review [14] of prospective clinical efficacy studies of acute, uncomplicated *P. vivax* malaria mono-infections treated with primaquine published between 1 January 2000 and 26 July 2024 in any language. Studies were eligible for the individual patient data meta-analysis if they were randomised therapeutic trials and prospective cohort studies published between 1 January 2000 and 29 September 2022 inclusive, with a minimum active follow-up of 42 days that recorded methaemoglobin data (at baseline and at least once in the first week of follow-up between day 5 and day 9) following daily primaquine administration given over multiple days. Two clinical trials published between 30 September 2022 and 26 July 2024 were not included in the analysis due to time constraints in obtaining approval and standardising these data. Only individuals with normal G6PD levels (i.e., $\geq$30% G6PD activity or a negative qualitative test) were included in the analysis. In individuals with G6PD deficiency, primaquine administration does not lead to the same methaemoglobin increases ...." (Methods, 1st paragraph)

Comment #2

We agree with Reviewer #3's comments regarding clarification that the aim of this study is to examine day 7 methemoglobin at the population level (rather than at the individual level). Please revise accordingly.

We have now revised to the manuscript to state "population-level" or "at the population level" throughout the manuscript. This includes text in:

- Abstract, Background, last sentence (e.g., "We undertook a systematic review and individual patient data meta-analysis to examine the utility of methaemoglobin as a population-level surrogate endpoint for 8-aminoquinoline antihypnozoite activity to prevent *P. vivax* recurrence.)
- Abstract, Conclusions, last sentence
- Author Summary, Why was this study done?, 4th bullet point
- Discussion, 2nd paragraph, last sentence
- Discussion, last paragraph, 1st sentence

Comment #3

ABSTRACT

1) Please combine the Methods and Findings sections into one section.

We have now combined both into one section.

2) l.71: Please define It at first use.

We have now clarified this throughout the manuscript.

3) l.71: “G6PD-normal patients” – please note that we prefer the use of patient-centered language (i.e. “patients with normal G6PD levels”). Please revise throughout the main manuscript accordingly.

We have revised the text to patient centred language, in the Abstract, Methods and Findings, 2nd sentence - "... patients with normal glucose-6-phosphate dehydrogenase (G6PD) activity enrolled ..."

4) l.76, please change to “P. vivax recurrence”.

'It’ has now been revised to "... in the risk of *P. vivax* recurrence ..." (Abstract, Methods and Findings, 2nd paragraph, 4th sentence).

5) l.76: Please define ‘CI’ at first use.

‘It’ has now been revised to "... 95% confidence interval [CI] = [0.57, 0.86]; ..." (Abstract, Methods and Findings, 2nd paragraph, 4th sentence).

6) l.76: We suggest changing the reporting to (i.e., removing the equal sign between "95% CI" and the values): (adjusted hazard ratio = 0.70; 95% CI [0.57, 0.86]; p = 0.0005). Please revise accordingly throughout the main manuscript.

We have now presented the confidence interval as suggested throughout the text.

7) In the last sentence of the Abstract Methods and Findings section, please describe the main limitation(s) of the study's methodology.

We have now added the following to the final sentence: "Study limitations include the inability to distinguish between recrudescence, reinfection, and relapse in *P. vivax* recurrences."

Comment #4

AUTHOR SUMMARY

Thank you for providing the Author Summary. Please note that the Author Summary should immediately follow the Abstract in your revised manuscript and not be submitted as a separate file.

1) The last bullet point under ‘Why was this study done?’ should become the first bullet point under ‘What did the researchers do and find?’.

The last bullet point under "Why was this study done?" has now been moved to the first bullet point under "What did the researchers do and find?".

2) “all available methaemoglobin and clinical data” – We suggest softening this statement.

We have rephrased the sentence to "We systematically reviewed and pooled the available methaemoglobin and clinical data from patients with *P. vivax* malaria treated with primaquine over the last 20 years." (Author Summary, What did the researchers do and find?, 2nd bullet point).

3) Please change to: Patients with higher methaemoglobin levels a week after starting primaquine treatment appear to have a lower risk of recurrence.

We have revised the sentence as suggested (Author Summary, What do these findings mean?, 1st bullet point).

4) Please change to: Day 7 methaemoglobin levels can potentially be used as a proxy for later vivax recurrence in exploratory trials, making it more efficient (e.g., fewer volunteers and resources required) to determine whether new drugs or regimens are effective.

We have revised the sentence as suggested (Author Summary, What do these findings mean?, 2nd bullet point).

5) In the final bullet point of 'What Do These Findings Mean?', please include the main limitations of the study in non-technical language.

We have added an additional bullet point under "What do these findings mean?" as follows: "Study limitations include the inability to determine whether *P. vivax* recurrences are due to treatment failure, reinfection, or relapse."

Comment #5

INTRODUCTION

l.129: “all available data” - We suggest softening this.

We have now removed the word 'all' from the sentence (Introduction, last paragraph, last sentence).

Comment #6

METHODS AND RESULTS

1) Figure 1: Please define ‘P. vivax’ in the figure title.

We have revised the figure title to "Directed acyclic graph showing our hypothesised causal relationships between primaquine-induced changes in blood methaemoglobin concentrations and *Plasmodium vivax* relapse." (Figure 1)

2) l.273: Please change the heading to “Results”.

We have revised the heading to 'Results'.

3) Figure 3: In the figure caption, please indicate the meaning of the bars and whiskers (e.g. median plus first quartile and third quartile).

We have revised the figure description: "... In a boxplot, the box shows the interquartile range with a line for the median, and the whiskers extend to data points within 1.5 times the interquartile range, highlighting potential outliers. ..." (Figure 3)

4) l.325, please change to: The highest levels were observed among adolescent patients.

We have corrected the typo to: "The highest levels were observed among adolescent patients." (Results, 4th paragraph, 3rd sentence)

5) ll.325-326: “Younger and older patients had lower primaquine-induced methaemoglobinaemia (Fig S5).” – If possible, could you define "younger" and "older" by specifying an age (e.g., <5 years and >60 years)?

We have made the figure title more specific: "Forest plot of the relationship between day 7 methaemoglobin levels and the risk of vivax recurrence".

However, we categorising age might imply that the data can provide precise information, which they do not in this context. Therefore, we deliberately use approximate language and convey the information through a visual representation of a smooth curve.

6) Figure 4: In the figure caption, please indicate the meaning of the bars, whiskers, and dots. For the top graph, please add a unit for the values on the x-axis. In addition, please describe what is shown in the two different graphs, especially the top graph.

We have added to the figure description: "In a boxplot, the box shows the interquartile range with a line for the median, and the whiskers extend to data points within 1.5 times the interquartile range, highlighting potential outliers (dots)." (Figure 4)

We have also added a unit for the horizontal axis (Figure 4).

We have also clarified the figure description in the top and bottom panels: "... Top panel highlights comparisons between the dose groups and recurrence status using percentile summaries for day 7 methaemoglobin levels. Bottom panel provides a more detailed view of individual variability in day 7 methaemoglobin levels across different daily primaquine doses received and recurrence statuses. ..." (Figure 4)

7) Figure 5: Please define 'CI' and 'N' in the figure description. We suggest changing the figure title to be more specific. Please consider avoiding the use of red and green in order to make your figure more accessible to those with colour blindness.

We have added "CI confidence interval, N number of patients." to the figure description and changed the arrow colours to blue and orange (Fig 5).

8) Table 1: Please change the characteristic ‘Male’ to ‘Sex, Male’. We suggest changing the characteristic ‘Age (years)’ (the first one) to ‘Median Age (years)’ and ‘Primaquine daily dose (mg/kg)’ to ‘Median Primaquine daily dose (mg/kg)’. Please remove ‘ACT artemisinin-based combination therapy’ from the list of abbreviations since it is not used in the table.

We have revised Table 1 as suggested.

Comment #7

DISCUSSION

1) l.391, please change to: “…we confirmed…”

We have revised the phrase as follows: "... we confirmed that ..." (Discussion, 1st paragraph, 1st sentence)

2) l.436: Please temper claims of primacy of results by stating, "to our knowledge" or something similar.

We have added the phrase to the sentence: "Although methaemoglobin is a surrogate endpoint for adherence to treatment, to our knowledge, these results are not driven by adherence (90% of patients had fully supervised treatment)." (Discussion, 4th paragraph, 5th sentence)

3) l.441: Please define ‘DHA-piperaquine’ at first use.

We have added to the sentence: "... dihydroartemisinin (DHA)-piperaquine, ..." (Discussion, 4th paragraph)

Comment #8

REFERENCES

1) Where website addresses are cited, please specify the date of access using use the word “accessed” (e.g. [accessed: 10/04/2024]).

We have included the access date as suggested (References 16, 25, 41).

2) Please ensure that journal name abbreviations match those found in the National Center for Biotechnology Information (NCBI) databases ([https://apc01.safelinks.protection.outlook.com/?url=http%3A%2F%2Fwww.ncbi.nlm.nih.gov%2Fnlmcatalog%2Fjournals&data=05%7C02%7Cifadilah%40oucru.org%7C27d74a9f0914468365c908dcb6132655%7C22514748bbda4cb89795bfa3fbeec018%7C0%7C0%7C638585440591337054%7CUnknown%](https://apc01.safelinks.protection.outlook.com/?url=http%3A%2F%2Fwww.ncbi.nlm.nih.gov%2Fnlmcatalog%2Fjournals&data=05%7C02%7Cifadilah%40oucru.org%7C27d74a9f0914468365c908dcb6132655%7C22514748bbda4cb89795bfa3fbeec018%7C0%7C0%7C638585440591337054%7CUnknown%7CTWFpbGZsb3d8eyJWIjoiMC4wLjAwMDAiLCJQIjoiV2luMzIiLCJBTiI6Ik1haWwiLCJXVCI6Mn0%3D%7C0%7C%7C%7C&sdata=7Lo1t8K8BYuWiAVr4VC4NI9mmxvaELcf9QNw%2FJm7qPc%3D&reserved=0)), and are appropriately formatted and capitalised. For example, “The American Journal of Tropical Medicine and Hygiene” in reference [2] should be “Am J Trop Med Hyg”.

We have corrected the journal name abbreviations, as suggested (References).

3) Please revise the supporting information references according to the comments above.We have corrected the journal name abbreviations, as suggested (References).

Comment #9

SUPPLEMENTARY MATERIAL

1) Thank you for providing the completed PRISMA-IPD checklist. Please replace the page numbers with paragraph numbers per section (e.g. "Methods, paragraph 1"), since the page numbers of the final published paper may be different from the page numbers in the current manuscript.

We have revised the PRISMA-IPD checklist as suggested (Table A in Supporting Information).

2) In the published article, supporting information files are accessed only through a hyperlink attached to the captions. For this reason, you must list captions at the end of your manuscript file. You may include a caption within the supporting information file itself, as long as that caption is also provided in the manuscript file. Do not submit a separate caption file.

When SI files are contained with a single file:

Please label the file as ‘S1 Supporting Information’.

Please apply alphabetical labelling to each table and figure contained within the S1 file. For example, ‘Fig A’ to ‘Fig Z’ and ‘Table A’ to ‘Table Z’.

Plain text does not need to be labelled and can just be given a title as necessary. For example, ‘Statistical Analysis Plan’.

Please cite tables/figures as ‘Fig A in S1 Supporting Information’ and/or ‘Table A in S1 Supporting Information’, for example.

Please cite plain text as, ‘Statistical Analysis Plan in S1 Supporting Information’, for example.

When SI files are uploaded as separate files:

Please label tables as ‘S1 Table’ (so on) and figures as ‘S1 Fig’ (and so on).

Any additional documents (protocols/analysis plans etc.) can be labelled as ‘S1 Protocol’, for example. Please cite items as exactly as labelled.

We have added a list of captions at the end of the manuscript, renamed the SI file, updated the caption labels throughout the manuscript and SI file, and revised how they are cited in the text.

**Reviewer #2**

Comment #1

The authors have addressed my points

Michael Dewey

Author response

We thank the reviewer for his valuable feedback, which has greatly contributed to the improvement of our manuscript.

**Reviewer #3**

Comment #1

In the revised version of the manuscript the authors have largely addressed my concerns. However, I think it should be made evident in the abstract and ideally also in the manuscript title that the aim of this study is to explore day 7 methemoglobin as a population-level (rather than individual-level) marker of primaquine anti-hypnozoite activity.

Author response

We have now emphasised this throughout the manuscript as suggested. We thank the reviewer for their helpful comments.

**Reviewer #5**

Comment #1

I consider that the points I raised after reading the first version of the submitted article were adequately addressed by the authors. In particular the clarification of the breakdown of the selected studies. consequently, I recommend the publication of this article in PLoS Med.

Author response

We thank the reviewer for their insights that have greatly contributed to improving our manuscript.
